# Primary and Secondary Grzybowski’s Generalized Eruptive Keratoacanthoma: A New Perspective on Management, Clinical Features, and Prognosis

**DOI:** 10.1111/ijd.70063

**Published:** 2025-09-11

**Authors:** Nicholas Florin Kormos, Carina Maria Petrenciu, Alin Stefan Vizitiu, Raluca Ghețe, Umer Nadir, Adrian Lucian Baican, Stanislav N. Tolkachjov

**Affiliations:** ^1^ Dermatology Iuliu Hațieganu University of Medicine and Pharmacy Cluj‐Napoca Romania; ^2^ Epiphany Dermatology DallasLewisville Texas USA; ^3^ Division of Dermatology Baylor University Medical Center Dallas Texas USA; ^4^ Department of Dermatology University of Texas at Southwestern Dallas Texas USA; ^5^ Texas A&M University College of Medicine Dallas Texas USA

**Keywords:** Ferguson‐Smith syndrome, generalized eruptive keratoacanthoma, Grzybowski’s syndrome, skin cancer, squamous cell carcinoma, Witten and Zak syndrome

## Abstract

Grzybowski's generalized eruptive keratoacanthoma (GEKA) is a rare variant of keratoacanthomas, characterized by hundreds to thousands of lesions, accompanied by pruritus, mucosal involvement, and comorbidities. Our aim was to analyze the clinical presentation, associated comorbidities, treatment strategies, and outcomes of GEKA. We conducted a literature review of all published cases between 1950 and 2024, following the Preferred Items for Systematic Reviews and Meta‐Analyses (PRISMA) reporting guidelines. A total of 143 articles were screened; 58 were included, yielding 64 cases. Of these, 24 were associated with severe comorbidities, while 40 were not. Cases without associated conditions were more likely to exhibit a worse prognosis, greater therapeutic resistance, and distinct clinical features. In contrast, cases associated with malignancy, systemic diseases, or other comorbidities tended to show better treatment responses and fewer complications. Pruritus remained the predominant symptom in both groups, with similar lesion morphology. The rarity of GEKA leads to a paucity of literature. Reporting bias and limited cases may lead to less generalizability. Based on our review and considering the differences in clinical presentation, demographics, associated comorbidities, complications, and prognosis, GEKA may be classified as primary or secondary to a malignancy, systemic disease, or other comorbidity, encouraging clinicians to have a high index of suspicion during evaluation and treatment for an underlying trigger.

**Abbreviations**
GEKA: Grzybowski's generalized eruptive keratoacanthoma
HPV: human papillomavirus
KA: keratoacanthoma
PRISMA: Preferred Items for Systematic Reviews and Meta‐Analyses
TTD: time to diagnosis

## 1 Introduction

Keratoacanthomas (KA) are a common type of cutaneous tumor that commonly present as solitary lesions. However, multiple KAs can simultaneously arise during a short timeframe [1]. Three types of variants have been described: Grzybowski's, Ferguson‐Smith, and the Witten and Zak type [2]. Currently, these variants do not have a clear etiology described in the literature, even though associations with other chronic diseases and neoplasms have been observed.

Grzybowski's generalized eruptive keratoacanthoma (GEKA) is a rare variant of multiple KAs, affecting mainly the skin and the mucous membranes [3]. It was first described in 1950 in a 57‐year‐old male who initially developed a single lesion which developed into a generalized eruption of small, enlarging papular tumors with a central umbilication, and after 4–8 months, necrosis occurred with scarring [4, 5].

Since then, another 63 cases have been reported and published [1, 2, 3, 4, 5, 6, 7, 8, 9, 10, 11, 12, 13, 14, 15, 16, 17, 18, 19, 20, 21, 22, 23, 24, 25, 26, 27, 28, 29, 30, 31, 32, 33, 34, 35, 36, 37, 38, 39, 40, 41, 42, 43, 44, 45, 46, 47, 48, 49, 50, 51, 52, 53, 54, 55, 56, 57, 58]. Furthermore, in 2014, a review proposing diagnosis and treatment criteria was published, showcasing unique characteristics and processes specific to this disease [59]. Our aim in this study is to evaluate the currently published cases of GEKA, propose and validate a new clinical classification based on its association with major comorbidities, and describe clinical features, disease complications, and treatment response.

## 2 Materials and Methods

**FIGURE 1:**
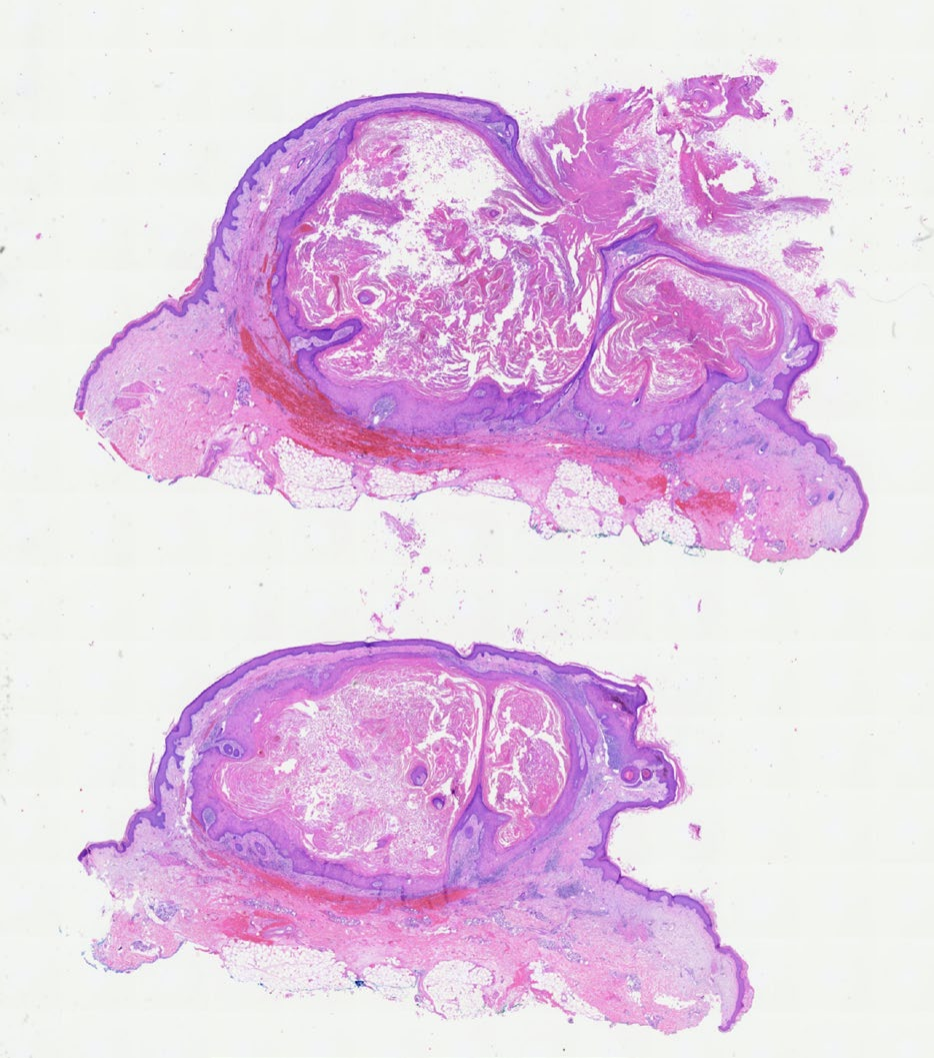
Hematoxilin‐eosin staining of a typical lesion revealing a crateriform lesion with a keratin plug, hyperkeratosis, acanthosis, no significant changes of the dermis (magnification ×2).

PubMed was searched for articles relating to GEKA published between 1950 and December 15, 2024. The study selection followed the Preferred Items for Systematic Reviews and Meta‐Analyses reporting guidelines (PRISMA); thus, all articles underwent two stages of screening, based on title/abstract screening and full‐text review. Inclusion criteria consisted of all published case reports and case series of patients meeting the diagnostic criteria for GEKA, specifically the Grzybowski type, regardless of the article's original language. Overall, 143 articles were screened, and 58 articles were included, yielding 64 patients (Figure S1). Variables, including demographics, medical history, GEKA evolution from baseline to follow‐up, course of treatment, presumed cause, and histopathologic reports/images (Figure 1), were collected.

GEKA was defined as primary when no known causative agent or comorbidity was identified or reported before, during, or after the GEKA diagnosis by the original authors. Secondary GEKA was classified as any GEKA diagnosis arising in the setting of chronic medical conditions that had severe systemic effects.

## 3 Results

### 3.1 Clinical Features and Diagnostic Criteria

**FIGURE 2:**
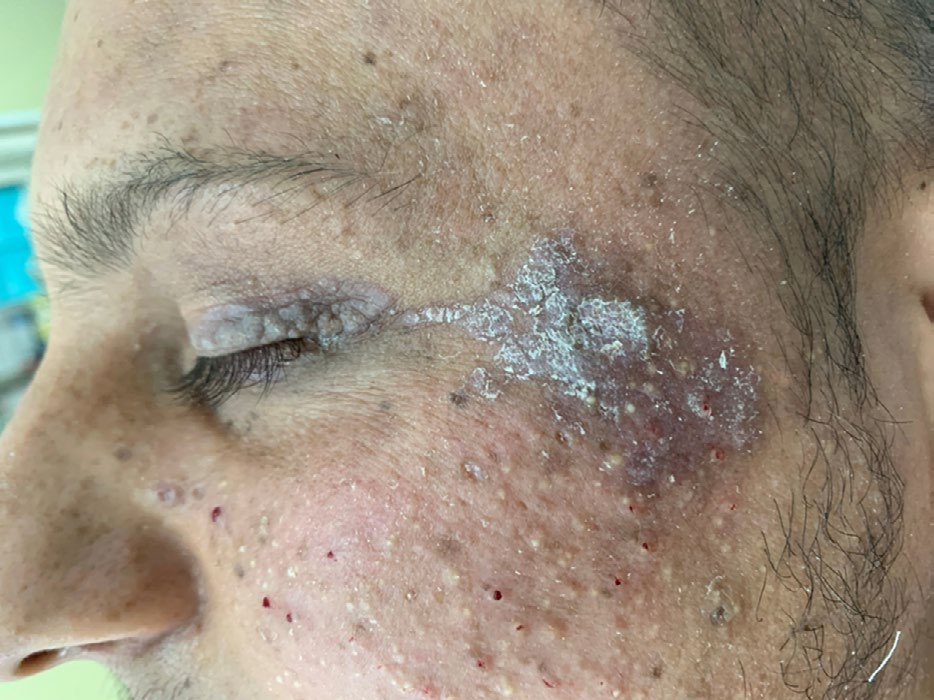
“Mask sign” present on the face of a male patient, with multiple papules forming a distinctive mask‐like appearance, which can also involve the eyelids and evolve into ectropion.

**FIGURE 3:**
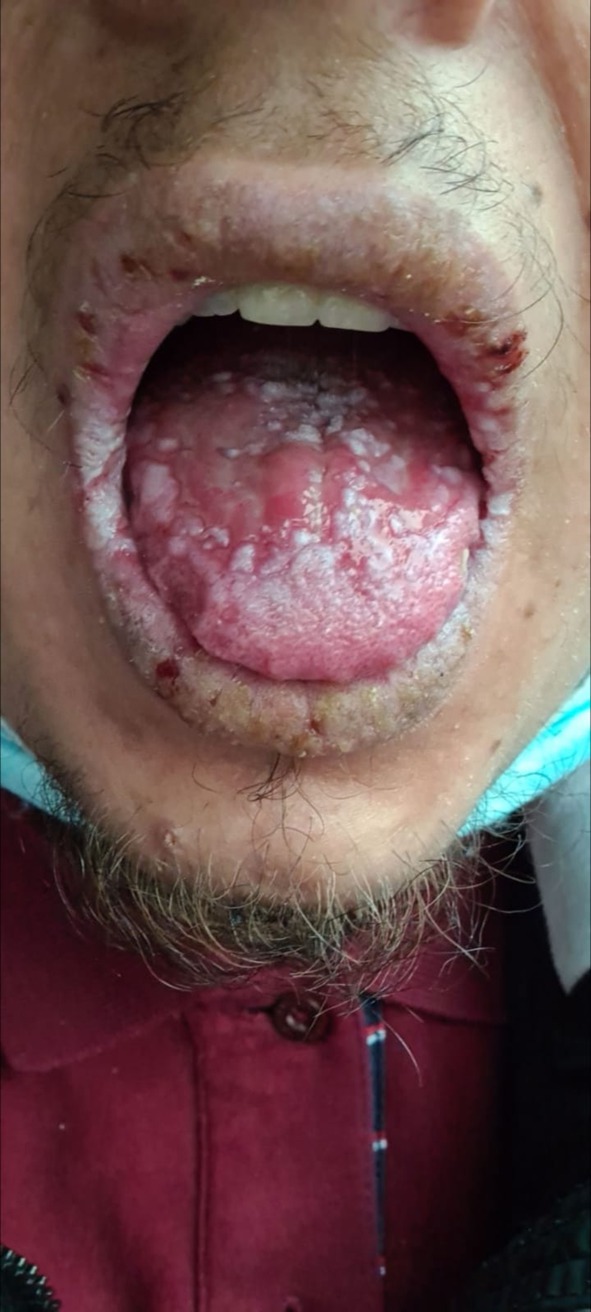
Oral mucosal ulcerations with multiple papules.

Among all cases of GEKA, common clinical criteria included: the sudden appearance of hundreds to thousands of rapidly growing follicular lesions ranging from 1–3 mm papules to 10–12 mm dome‐shaped nodules, with central keratotic plugging and umbilication (for lesions over 5 mm) [9, 19, 21, 31, 40]. Some cases developed larger lesions, simulating solitary KA [19]. Lesions may coalesce and also köbnerize [11, 37]. The multiple sizes of these lesions were described in seven primary GEKA cases and three secondary GEKA cases. Clinical progression is fairly expedited with an eruption of KAs appearing weeks to months after the first lesion [33, 45, 59]. Pruritus is usually present [21, 40, 59]. Facial involvement is common, resulting in masked‐like facies (Figure 2) and ectropion [3, 35]. The oral mucosa is also commonly involved (Figure 3) [13, 45, 48]. Histopathological findings from these cases are similar to those of solitary keratoacanthomas. Furthermore, diagnostic criteria for GEKA require similarity to solitary keratoacanthomas on histopathology [19, 31, 59].

Primary GEKA cases were described as forming coalescing plaques in three cases. One case presented as plaques formed from papules, another from nodules, and a third with variable morphologies of all sizes [17, 24, 33]. Another two cases of primary GEKA presented as keratoacanthomas on an erythematous background. These clinical features (coalescing lesions and background erythema) were absent in secondary GEKA cases [22].

### 3.2 Pathogenesis

GEKA has associations with various comorbidities including malignancies, autoimmune diseases, renal failure, uncontrolled hypertension, syphilis, anemia, and others [23, 24, 37, 48, 50]. Furthermore, there are several medications with associations to GEKA, including leflunomide [57], sorafenib [38, 51], and nivolumab [52]. Chemical, traumatic, viral, and immunologic etiologies have also been proposed. An association with malignancy has been proposed, but case scarcity prevented any statistical link to the findings [57].

About one‐third of all GEKA cases were reported in patients with severe comorbidities and developed GEKA after they were diagnosed with their respective comorbidities. Nine cases had a history of malignancy, seven cases of autoimmune diseases, eight with hypertension, six with severe renal impairment, one with severe depression, one with morbid obesity, one with alcoholic liver cirrhosis, and one with ischemic heart disease (Table 1).

**TABLE 1:** Comorbidities of patients with secondary GEKA.

	Author	Malignancy	Autoimmune disease	Renal involvement	Other	Medication	HTN
1	M. N. Abbas et al.	HCC	—	—	Hepatitis C, CLD	**S**	YES
2	H. Mascitti et al.	—	—	—	Depression, HPV39	—	YES
3	P. W. Jaber et al.	—	—	—	Morbid obesity, DM	—	—
4	F. Filippi et al.	—	RA	—	—	—	YES
5	D. H. Chu et al.	**Breast cancer**	Psoriasis	—	—	T	YES
6	R. Havenith et al.	—	—	**TKD** , IMN, RT	Hepatopathy, FVL	—	YES
7	N. Haas et al.	Colon carcinoma	—	—	—	—	—
8	Q. Xu et al.	—	Vitiligo	—	Prurigo nodularis	—	—
9	J. Norgauer et al.	—	—	DN	DM, ALD, CLD	—	—
10	J. Norgauer et al.	—	SSc	—	—	—	—
11	S. Munemoto et al.	—	SS, Elevated ANA	—	—	—	—
12	R. Winkelmann et al.	—	—	MNS, ARF	—	—	YES
13	B. L. Snider et al.	**OvAC**	—	—	Muir‐Torre syndrome^a^	—	—
14	C. V. Washington Jr. et al.	—	—	**RT** , CKD	—	**P**	—
15	M. W. Dessoukey et al.	—	SLE	SLE	—	P	—
16	H. Jantzem et al.	**ccRCC**	—	ccRCC	Gout	S	YES
17	S. Feldstein et al.	Pancreatic cancer	—	—	—	**N**	—
18	H. Jolly Jr. et al.	—	SLE	—	**DS, SD**	—	YES
19	E. Willinger et al.	Rectal carcinoma	—	—	CD 25—D	—	—
20	K. Davies et al.	—	—	—	IHD	—	—
21	G. Weber et al.	FTC, SCL	—	—	—	—	—
22	P. Cabotin et al.	**NHL**	—	—	—	—	—
23	W. Tidwell et al.	—	SLE, RA, sarcoidosis	—	DM, COPD	**L**	YES
24	P. Ilaria et al.	—	—	—	DM, obesity	**C**	YES

*Note:* Bolded and underlined comorbidities or medications were considered causative by the reporting authors.

Abbreviations: ALD = alcoholic liver disease; ANA = antinuclear antibodies; ARF = acute renal failure; C = Comirnaty; ccRCC = clear cell renal adenocarcinoma; CD 25—D = CD 25 deficiency; CLD = chronic liver disease; DM = diabetes mellitus; DN = diabetic nephropathy; DS = diverticular sigmoiditis; FTC = fallopian tube carcinoma; FVL = factor V Leiden mutation; HCC = hepatocellular carcinoma; HTN = hypertension; IHD = ischemic heart disease; IMN = idiopathic membranous nephropathy; L = leflunomide; MNS = malignant nephrosclerosis; N = nivolumab; NHL = non‐Hodgkin lymphoma; OvAC = ovarian adenocarcinoma; P = prednisone; RA = rheumatoid arthritis; RT = renal transplant; S = sorafenib; SARC = sarcoidosis; SCL = stem‐cell leukemia; SD = sigmoidectomy; SLE = systemic lupus erythematosus; SS = Sjogren's syndrome; SSc = systemic scleroderma; T = tamoxifen; TKD = terminal kidney disease.

^a^ Suspected 36–58.

Given the multiple comorbidities reported in the literature, it is difficult to determine if the cause is related to the disease itself or to the treatment associated with the pathologies.

### 3.3 Patient Demographics

In total, 64 cases were extracted from the PubMed database, out of which 40 patients were found to have minimal to no comorbidities while 24 of them were found to have severe comorbidities, with a ratio of approximately 2:1 [1, 2, 3, 4, 5, 6, 7, 8, 9, 10, 11, 12, 13, 14, 15, 16, 17, 18, 19, 20, 21, 22, 23, 24, 25, 26, 27, 28, 29, 30, 31, 32, 33, 34, 35, 36, 37, 38, 39, 40, 41, 42, 43, 44, 45, 46, 47, 48, 49, 50, 51, 52, 53, 54, 55, 56, 57]. The mean age of patients diagnosed with GEKA is 59.37 years, with a large majority of patients presenting between 46 and 71 years old (range: 23–94) [15, 50]. The vast majority of patients were Caucasian (*n* = 59, 92%), while the rest were of Asian (*n* = 3, 5%) and African (*n* = 2, 3%) descent. A 3:2 female predominance was observed, with 38 cases being female and 26 male. No correlation with smoking was observed (Table S1).

The primary GEKA group had a mean age of 60 years with 38 Caucasian patients, one Asian patient, and one Black patient. A history of smoking was only mentioned in three cases. Eight patients were described as having significant UV exposure. Four cases were previously diagnosed with skin cancer: two cases with squamous cell carcinoma, one with basal cell carcinoma, and one unspecified. A single case had herpes zoster 8–10 weeks before GEKA [7]. Other cases did not report infections.

The secondary GEKA group had a mean age of 57.5 years with 21 Caucasian patients, two Asian patients, and one Black patient. No smoking history was mentioned, and no significant sun exposure was described. No previous skin cancers were reported. Two cases of human papillomavirus (HPV) presence in the lesions were described; however, testing in others may not have been reported [46].

### 3.4 Initial Presentation and Symptoms

Initial clinical signs for primary GEKA cases were eruptive papules, nodules, or tumors of various sizes (*n* = 14, 35%), pruritus (*n* = 7, 17.5%), eruptive papules (*n* = 6, 15%), and isolated tumors (*n* = 2, 5%). For secondary GEKA cases, pruritus was the most common first sign (*n* = 7, 29%), followed by eruptive lesions of various sizes (*n* = 5, 21%), papular eruptive tumors (*n* = 2, 8%), and isolated tumors (*n* = 2, 8%). No significant differences were observed in clinical presentation between these two subtypes.

The mean time to diagnosis (TTD) was 1.83 years for primary GEKA and 1.25 years for secondary GEKA, varying based on initial signs. Cases with eruptive tumors of various sizes had a mean TTD of 3.93 years for both variants vs. 1.02 years for pruritus as a first sign (*p* = 0.021). Cases with 1–3 mm papules had a mean TTD of 0.64 years, but due to a small sample size, there was no statistically significant difference in TTD compared with cases of pruritus (*p* = 0.346) or larger eruptive tumors (*p* = 0.075).

Pruritus is the main symptom accompanying GEKA. Primary GEKA cases reported a higher incidence of pruritus (*n* = 30, 75%), with only six cases (15%) considered severe. A majority of cases with reported pruritus presented in the form of eruptive papules or tumors (*n* = 18, 60%). For secondary GEKA, pruritus was described less commonly (*n* = 10, 42%), and only one case was considered severe. Pruritus is significantly more common in primary GEKA than in secondary GEKA (*p* = 0.008), but interestingly, the most common initial sign for secondary GEKA was pruritus (*n* = 7, 70%) prior to lesions developing. Only one case of secondary GEKA was represented by eruptive papules or tumors as the initial clinical sign, proving a statistically significant difference in presentation between primary (eruptive lesions) and secondary (pruritus) GEKA (*p* = 0.024, Fisher's exact).

### 3.5 Histopathology

The histopathology of GEKA lesions is described as similar to solitary AKs, including a central keratin‐filled crater, acanthosis, glassy eosinophilic hyaline keratinocytes [30, 45], horn pearls with central keratinization [23], and an epidermal collar (buttressing) [19]. Inflammatory cells are usually present in large numbers, with lymphocytes and histiocytes usually present [10, 34, 54]. Smaller papules may present atypical features consistent with an incipient KA and may pose a diagnostic challenge if no mature lesions are biopsied [40]. Biopsies performed under treatment, even if the treatment is ineffective, may lead to false negative results as the pathologist may not recognize the lesions as keratoacanthomas [58]. Overlaps with hypertrophic lichen planus histopathology have also been reported [33].

### 3.6 Complications

There are four main complications described with GEKA: ectropion, mask‐like facies, scarring, and oral involvement. Ectropion has even been reported to cause blindness in GEKA patients [2, 9]. Ectropion is more common in primary (*n* = 24, 60%) than in secondary (*n* = 6, 25%) GEKA (*p* = 0.007). The “mask sign” results from severe facial disfigurement and has a similar incidence in both forms of GEKA (*n* = 8, 20%), (*n* = 5, 21%). Scarring after the resolution of KAs is common and is more prevalent in primary (*n* = 15, 37.5%) than in secondary (*n* = 3, 13%) GEKA (*p* = 0.038). The involvement of the oral mucosa affected both primary (*n* = 20, 50%) and secondary (*n* = 9, 38%) forms, but without statistical significance.

Patients with primary GEKA had more complications than patients with secondary GEKA. Furthermore, more than one complication was observed in 40% of cases with primary GEKA vs. 25% of cases in secondary GEKA (*p* = 0.222); a single complication was present in 27.5% of cases with primary GEKA vs. 13% of secondary GEKA cases. Complication‐free disease courses were less common in primary GEKA (*n* = 13, 32.5%) compared to the cases of secondary GEKA (*n* = 15, 65%) (*p* = 0.019).

### 3.7 Treatment Options and Resolution

Currently recommended treatments include oral retinoids as first‐line, followed by methotrexate and cyclophosphamide as second‐ and third‐line treatments, respectively [59]. Other therapies that have been used in the literature include aminopterin, cimetidine, hydroxychloroquine, and cemiplimab [24, 28, 29, 45, 58]. Topical treatments have also been proposed with varying success rates [59]. Radiotherapy was used in two patients without success [23].

Outcomes from treatment were missing for two cases of primary GEKA and three cases of secondary GEKA (Table 2). From the remaining pool of cases, primary GEKA patients achieved complete resolution in 18% of cases, partial resolution in 18% of cases, and no response in 63% of cases. In secondary GEKA, 43% of patients achieved complete resolution (*p* = 0.043), partial resolution in 43% of cases (*p* = 0.043), and no resolution in 14% of cases (*p* < 0.001). Interestingly, primary GEKA has been reported to respond significantly better to acitretin and isotretinoin over other therapeutic options reported (*p* = 0.034). For secondary GEKA, the best therapeutic outcome can be achieved by treating the underlying comorbidity. After initiating treatment for underlying comorbidities, cutaneous lesions from GEKA responded to systemic and topical (4/5 cases) therapies.

**TABLE 2:** Therapeutic options described in primary and secondary GEKA.

Last reported therapy	Primary GEKA			Secondary GEKA		
	No response	Partial resolution	Full resolution	No response	Partial resolution	Full resolution
Acitretin (25–50 mg/day)	6	2	2	—	2	2
Isotretinoin (40–120 mg/day)	1	1	4	—	—	2
Etretinate (10–80 mg/day)	5	1	0	1	2	1
Methotrexate (15–20 mg/week)	2	—	—	—	1	1
Cyclophosphamide (100 mg/day)	1	2	1	1	—	—
5% 5‐FU^a^ (3–5 applications/week)	1	—	—	1	3	1^b^
Cyclosporine (2.5 mg/kg/day)	—	—	—	—	—	1
Hydroxychloroquine (200 mg/day)	—	—	—	—	1	—
Cimetidine (2.4 g/day)	—	1	—	—	—	—
Cryotherapy^c^	—	—	—	—	—	1
Topical CS	1	—	—	—	—	—
Vitamin A	1	—	—	—	—	—
None^d^	4	—	—	—	—	—
Cemiplimab	—	—	—	—	—	1
No therapy was effective	2	—	—	—	—	—
Total	24	7	7	3	9	9

*Note:* Reported dosage intervals are noted in parenthesis.

^a^ 5‐Flourouracil.

^b^ Combination of 5‐FU, imiquimod cream, and lapacho tea.

^c^ Select lesions were treated and the bulk resolved 4 months after nivolumab discontinuation.

^d^ Cases where patients refused therapy.

## 4 Discussion

In our review, we have observed two distinct forms of GEKA: primary/idiopathic GEKA or secondary GEKA either to a malignancy, systemic disease, or other comorbidity. These distinct manifestations include differing clinical presentations, outcomes, and treatments. Historically, GEKA has been hypothesized to be triggered by various clinical entities, the leading assumption being a viral trigger. However, current research and published cases do not support that hypothesis [44, 59]. Previous studies showed an association with malignancies, but the correlation was considered too weak and abandoned by subsequent authors [40, 50]. Our hypothesis is that the causes for GEKA in a majority of cases are still unknown, with a possible immunologic or genetic origin. Secondary cases seem to be strongly linked with their triggering disease and respond to treatment of the underlying comorbidity.

When considering the head and neck region specifically, we believe that both forms of primary and secondary GEKA lead to significant morbidity and quality of life impairment, with patients experiencing primary GEKA being more at risk of severe complications.

Among the three cases that had no response, one refused systemic treatment as she believed the therapy was causing the disease, one died a week after systemic therapy was initiated due to complications from a malignancy, and one received only topical 5‐fluorouracil [44, 49, 55].

All nine cases that had a history of malignancy developed GEKA after their initial diagnosis (Table 1) [38, 42, 44, 48, 51, 52, 53, 55, 57].

It is unclear if secondary GEKA is triggered by the medication involved in treating the initial comorbidities or related directly to the comorbidities themselves. Five cases of secondary GEKA with a therapeutic link have been published, but currently no definitive answer can be drawn on causality [38, 51, 52, 57, 58].

Multiple therapeutic options have been reported for GEKA, as noted in the results section; however, acitretin and isotretinoin have been most efficacious for primary GEKA in the literature. Cyclophosphamide is often used as a third‐line therapy for primary GEKA, and the use of alternative therapies such as cimetidine may be considered.

For secondary GEKA, topical 5‐fluorouracil may be initiated as first‐line therapy. Retinoids should be considered a second‐line therapy, and methotrexate as a third‐line therapy. Cyclosporine may be used for recalcitrant cases, depending on the comorbidities. Etretinate, although used in several published cases, was the least efficacious retinoid reported (Figure S2).

## 5 Limitations

Due to disease rarity, all reported cases were included. Reported data were inconsistent among cases; therefore, all relevant data were included to reduce bias as much as possible.

## 6 Conclusion

GEKA is a rare disease that is considered to have a variable outcome. The diagnosis is mainly clinical, with histopathological confirmation commonly used in assessment. We propose the classification of GEKA into primary and secondary, distinguished by the presence of underlying comorbidities, clinical presentation, complications, and therapeutic response. Systemic therapy with isotretinoin should be considered first‐line for primary GEKA. The main treatment goal for secondary GEKA should be the treatment of the underlying comorbidity, while cutaneous lesions may respond to topical 5‐fluorouracil.

Given the rarity of GEKA and variability in reporting, prospective data collection and standardized case reporting are essential. The pathogenesis of these entities is still unclear, and future research into the genetic contribution toward the development of the disease should be addressed. In the case of secondary GEKA, future research may look into triggering factors of systemic comorbidities and therapeutic response after treatment of these comorbidities.

## Consent

The patients in this manuscript have given written informed consent to publication of their case details.

## Conflicts of Interest

Dr. Tolkachjov is a speaker and investigator for Kerecis, Boehringer Ingelheim, and CASTLE Biosciences. No relevant conflicts of interest.

## Supporting information


**Supplementary Material:** ijd70063‐sup‐0001‐Supinfo.docx

## Data Availability

The data that support the findings of this study are available from the corresponding author upon reasonable request.
