# Supplementary material for: Primary and Secondary Grzybowski’s Generalized Eruptive Keratoacanthoma: A New Perspective on Management, Clinical Features, and Prognosis
Source: Int J Dermatol. 2025 Sep 11;65(3):456–63. doi: 10.1111/ijd.70063 (PMC12892160; doi:10.1111/ijd.70063)
Supplement: Supplementary file 1 — Supplementary Material: ijd70063‐sup‐0001‐Supinfo.docx [file IJD-65-456-s001.docx]

**Supplementary Material:** Primary and Secondary Grzybowski’s Generalized Eruptive Keratoacanthoma: A New Perspective on Management, Clinical Features, and Prognosis

**Figure S1.** PRISMA flow diagram of included studies


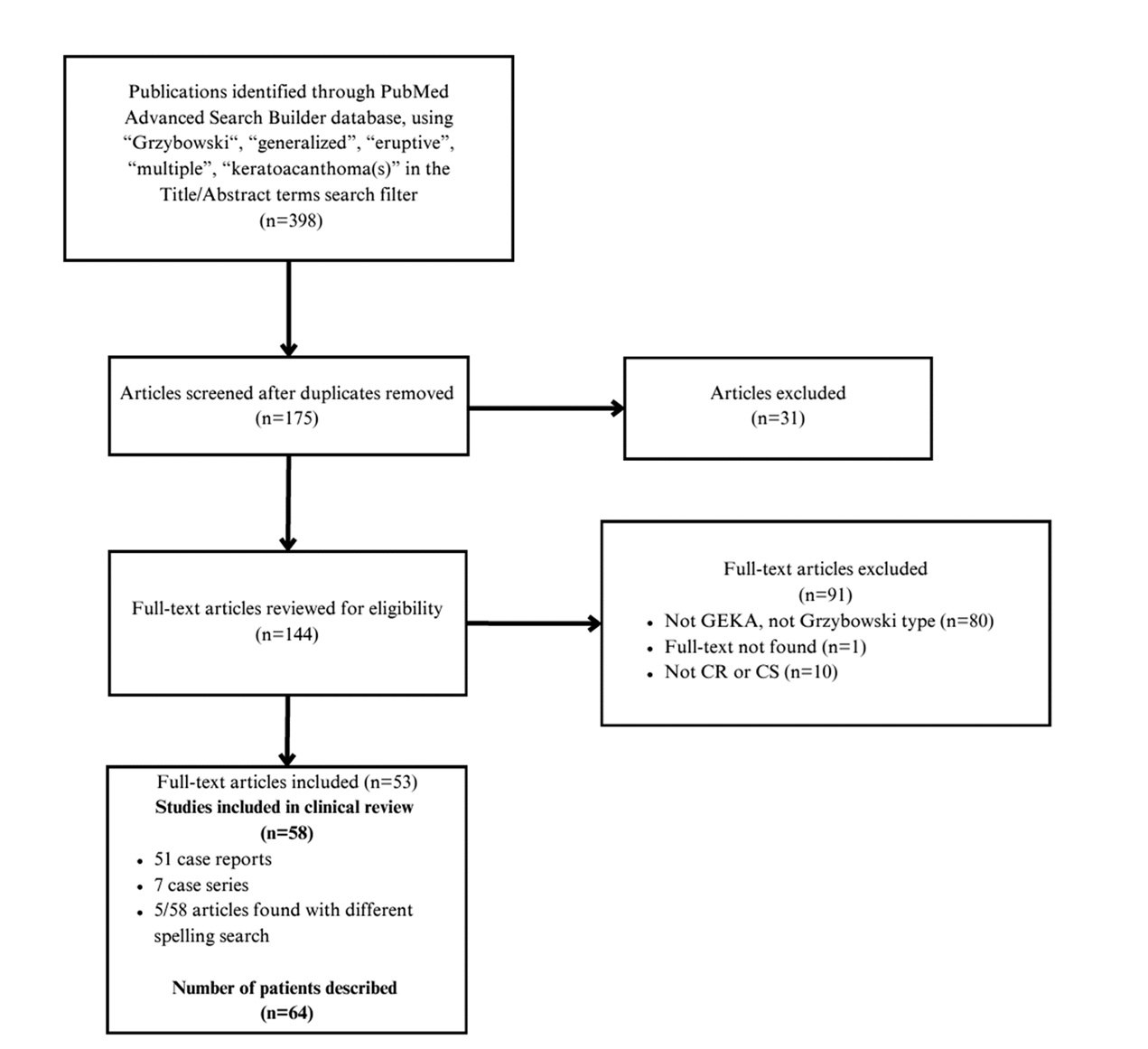


**Figure S2.** Treatment algorithm for Grzybowski’s generalized eruptive keratoacanthoma (GEKA)


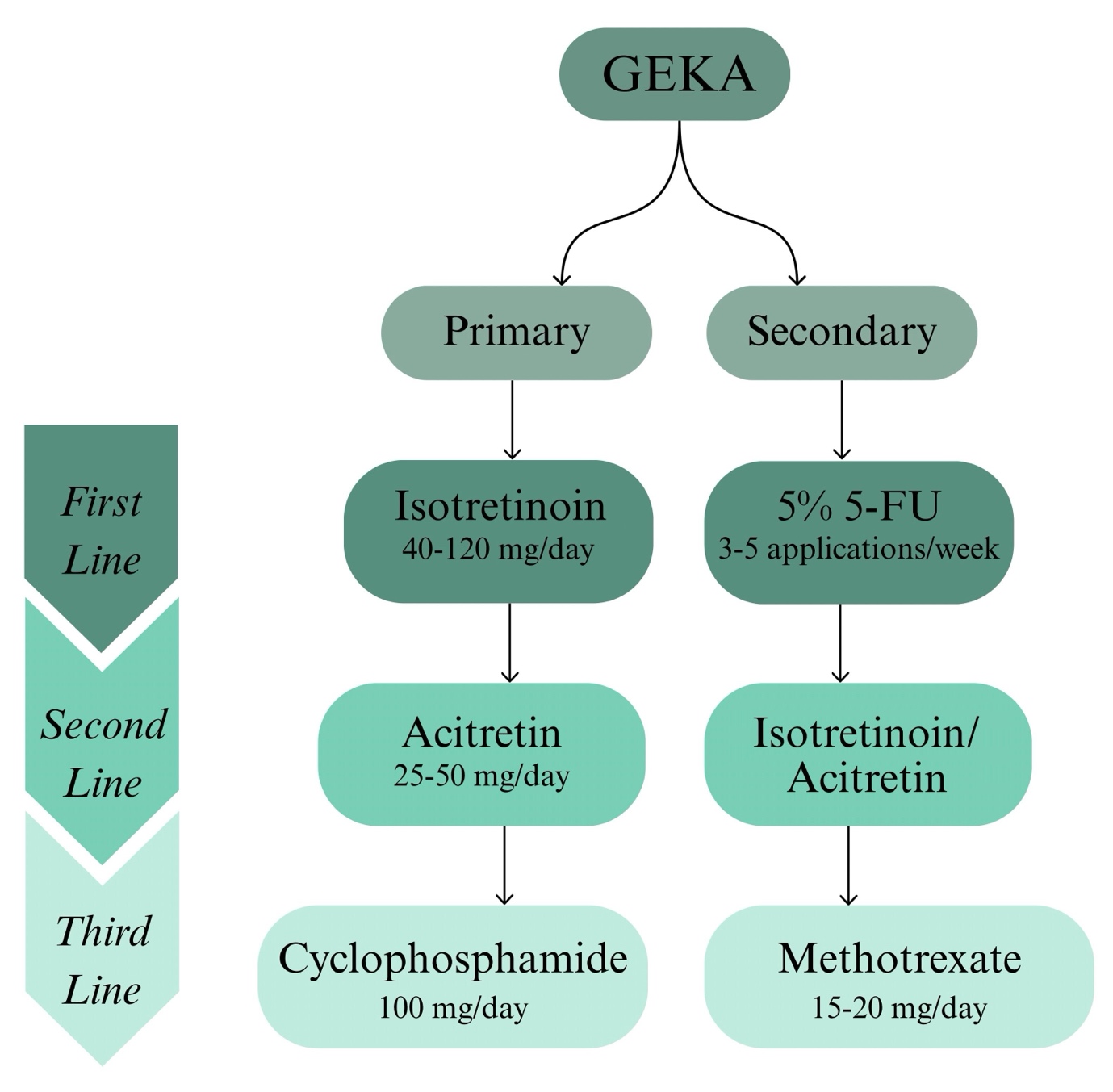


**Supplementary Table 1:** Demographic and Clinical Characteristics of Primary vs. Secondary GEKA Cases.

| **Characteristic** | **Primary GEKA (n = 40)** | **Secondary GEKA (n = 24)** |
| --- | --- | --- |
| **Mean age (years)** | 60 | 57.5 |
| **Sex (F:M)** | 11:9 | 5:3 |
| **Ethnicity** | 38 Caucasian, 1 Asian, 1 Black | 21 Caucasian, 2 Asian, 1 Black |
| **Smoking history reported** | 3 cases (7.5%) | None reported |
| **Significant sun exposure** | 8 cases (20%) | None described |
| **Previous skin cancer** | 4 cases (10%) | None reported |
| – Squamous cell carcinoma | 2 cases | – |
| – Basal cell carcinoma | 1 case | – |
| – Unspecified | 1 case | – |
| **Infectious history** | 1 case with herpes zoster | 2 cases with HPV detected in lesions |
| **Comorbidities** | None or minimal | All had ≥1 severe comorbidity |
